# Allen Brain Atlas-Driven Visualizations: a web-based gene expression energy visualization tool

**DOI:** 10.3389/fninf.2014.00051

**Published:** 2014-05-21

**Authors:** Andrew Zaldivar, Jeffrey L. Krichmar

**Affiliations:** ^1^Department of Cognitive Sciences, University of California, IrvineIrvine, CA, USA; ^2^Department of Computer Science, University of California, IrvineIrvine, CA, USA

**Keywords:** Allen Brain Atlas, data-driven documents, gene expression, neuroinformatics, data visualization

## Abstract

The Allen Brain Atlas-Driven Visualizations (ABADV) is a publicly accessible web-based tool created to retrieve and visualize expression energy data from the Allen Brain Atlas (ABA) across multiple genes and brain structures. Though the ABA offers their own search engine and software for researchers to view their growing collection of online public data sets, including extensive gene expression and neuroanatomical data from human and mouse brain, many of their tools limit the amount of genes and brain structures researchers can view at once. To complement their work, ABADV generates multiple pie charts, bar charts and heat maps of expression energy values for any given set of genes and brain structures. Such a suite of free and easy-to-understand visualizations allows for easy comparison of gene expression across multiple brain areas. In addition, each visualization links back to the ABA so researchers may view a summary of the experimental detail. ABADV is currently supported on modern web browsers and is compatible with expression energy data from the Allen Mouse Brain Atlas *in situ* hybridization data. By creating this web application, researchers can immediately obtain and survey numerous amounts of expression energy data from the ABA, which they can then use to supplement their work or perform meta-analysis. In the future, we hope to enable ABADV across multiple data resources.

## Introduction

Gene expression is the process by which a gene, a segment of DNA, is turned into a protein or RNA structure. When studying gene expression, researchers analyze changes in the expression of a particular gene or set of genes by quantifying the amount of its gene-specific transcript. The ability to measure and localize these gene-specific transcripts in the nervous system enables researchers to investigate a broad range of brain phenomena. Researchers use gene expression data in various ways, such as looking at profiles or patterns of expression across several genes, cross-species comparisons, searching for biomarkers, validating different data modalities, correlating gene expression to neuroanatomy, and other large-scale data analysis (Jones et al., 2009). Due to its broad application, many organizations have put together publicly accessible neuroinformatic resources comprised of massive amounts of gene expression data in order for other researchers to foster new discoveries.

The Allen Institute for Brain Science is one such research organization that created the Allen Mouse Brain Atlas (ABA), a growing collection of online public data sets that integrates extensive gene expression and neuroanatomical data complete with a suite of search and viewing tools (Hawrylycz et al., 2012; Sunkin et al., 2013). The ABA and its vast array of resources enabled researchers to develop new methods for investigating brain data. For instance, Liscovitch (Liscovitch et al., 2013) created FuncISH, a method to learn functional representations of any neural *in situ* hybridization (ISH) images by applying Gene Ontology categories with the genomic set of mouse neural ISH images available in the ABA. Another group also systematically explored high resolution ISH images contained in the ABA by using a data mining tool they developed called Hippo-ATESC (Automatic Texture Extraction from the Hippocampal region using Soft Computing), which helped detect neuropil-encoded genes in the hippocampus that are known for their involvement in synaptic structure and plasticity (Ugolotti et al., 2013). Ji et al. (2014) integrated resources from both the ABA and the recent Allen Mouse Brain Connectivity Atlas to systematically study the relationship between gene expression and structure-level brain connectivity by employing ensemble models for predicting brain connectivity. Altogether, the richness of the ABA resources helps researchers conduct scientific data analysis and discover new knowledge in neuroscience, accelerating our understanding of how the brain works.

Though many of these resources and applications provided by the ABA and other groups offer sophisticated ways of navigating across ABA's large database, these tools come with a steep learning curve. For example, many of ABA's tools such as their Brain Explorer application, though useful in some ways, limit the amount of genes and brain areas researchers can view at once. Furthermore, while the ABA provides documentation and tutorials on how to use their resources, some users may not want to devote their time in reading and understanding their overwhelming amount of features for retrieving a small amount of data.

The ABA provides programmatic access to their published data set for any user to perform any data retrieval and analysis beyond interfacing with their existing software. Some groups that have built software on top of the ABA through this programmatic service are typically motivated by a specific hypothesis-driven analysis (Ugolotti et al., 2013; Ji et al., 2014). Though beneficial, this type of software may prevent other users from using their software in the first place, as they might instead prefer software that enables explorative analysis. Thus, it may be useful to build new software applications that increase the flexibility of exploring the ABA.

Using the ABA application programming interface (API), we created a web-based, free access and open source application called the Allen Brain Atlas-Driven Visualizations (ABADV). ABADV generates simple-to-analyze visualizations of numerous mouse gene expression data across brain structures using Data-Driven Documents (D3), a Javascript library that uses data to drive the creation and control of visualizations in web browsers (Bostock et al., 2011). ABADV allows researchers to immediately obtain and survey mouse gene expression data using a variety of visualizations, which can then be used to supplement their work or perform meta-analysis. ABADV is available for anyone to try with no download required at: http://www.socsci.uci.edu/~jkrichma/ABADV/

In this article, we describe the details of how ABADV works and provide representative demonstrations with queries containing lists of genes that encode dopamine and serotonin in brain structures associated with the reward circuit (Nakamura, 2013; Russo and Nestler, 2013). We then compared and contrasted our results with the same query performed using Brain Explorer 2, a desktop application created by the Allen Institute for viewing their reference atlases and gene expression data in 3D (Sunkin et al., 2013). As large-scale data generation and integration continues to grow in modern neuroscience, web applications such as ABADV could potentially accelerate scientific discovery and technological progress, which may result in major clinical and economical benefits.

## Materials and methods

In this section, we describe the implementation of ABADV, a web-based application we created for visualizing expression energy data from the ABA. By combining the ABA API, a resource enabling programmatic access to the ABA dataset, with D3, a library that uses digital data to drive the creation and control of dynamic and interactive visualizations, ABADV offers a way to quickly identify prevalent expression from a long list of genes and brain structures. The source code for ABADV is available at: http://github.com/UCI-CARL/ABADV

### Allen mouse brain atlas

Data used by ABADV are retrieved from the ABA, a resource available through the ABA data portal (http://www.brain-map.org) (Lein et al., 2007; Jones et al., 2009; Sunkin et al., 2013). The ABA is a genome-wide 3D map of gene expression from 56-day-old male C57BL/6J mice strains (Lein et al., 2007; Jones et al., 2009; Sunkin et al., 2013). This map is comprised of high-resolution images from ISH data spanning across approximately 20,000 genes, generated by using non-radioactive, digoxigenin-labeled anti-sense riboprobes (Lein et al., 2007; Jones et al., 2009; Sunkin et al., 2013). The Allen Reference Atlas (Dong, 2008) made registration and alignment of this map with anatomical information possible, producing an integrated suite of sophisticated data search and visualization tools that help their users discover where each gene is expressed in the adult mouse brain (Ng et al., 2007, 2009; Lau et al., 2008).

In addition to the entire suite of search and visualization tools offered across the ABA, an API is available for programmatic access (Sunkin et al., 2013). The API core consists of three components: (1) Representational State Transfer (RESTful) Model Access that allows programmatic searching and retrieving of experimental data in JavaScript Object Notation (JSON), Extensile Markup Language (XML) and comma-separate values (CSV); (2) web services to download high-resolution images, computed gene expression statistics at the grid voxels level, reference atlas 3D models and ontologies, and programmatic access to search tools available in their user interface; and (3) demonstration applications using different parts of the API, complete with source code. Full documentation on the API and example applications can be found directly at http://www.brain-map.org/api/index.html

### Expression energy

The data retrieved by ABADV from the ABA API represents quantified gene expression energy values per 200 μm voxel of the mouse brain. To obtain these values, the Allen Institute developed an informatics data processing pipeline on top of their high-throughput RNA ISH data generation to enable the navigation, analysis and visualization of their massive dataset (Dang et al., 2007; Ng et al., 2007). To summarize, each image per experiment is divided and reconstructed into a 200 μm^3^ 3D grid. For each division, the number of expression pixels and expression pixel intensity were summed to collect pixel-based statistics such as the expression density (sum of expressing pixels divided by sum of all pixels in division) and expression intensity (sum of expression pixel intensity divided by sum of expression pixels). From these statistics, the ABA calculated the expression energy, defined as the sum of expression pixel intensity divided by the sum of all pixels in division. Expression energy is then computed for each brain structure delineated in the Allen Reference Atlas by unionizing grid voxels with the same 3-D structural label. Aligning the expression energy back to the coordinate space of the Allen Reference Atlas allows for cross-comparison of gene expression data between brain structures.

### Data-driven documents

ABADV visualized data by using D3, a Javascript library that uses digital data to drive the creation and control of dynamic and interactive web-based visualizations (Bostock et al., 2011). D3 works by binding input data to arbitrary document elements through the document object model (DOM) API. The DOM API enables collaboration of web technologies such as HyperText Markup Language (HTML) for page creation, Cascading Style Sheets (CSS) for aesthetics, JavaScript for web interaction, and Scalable Vector Graphics (SVG) for vector graphics (Bostock et al., 2011).

### Implementation

**Figure 1:**
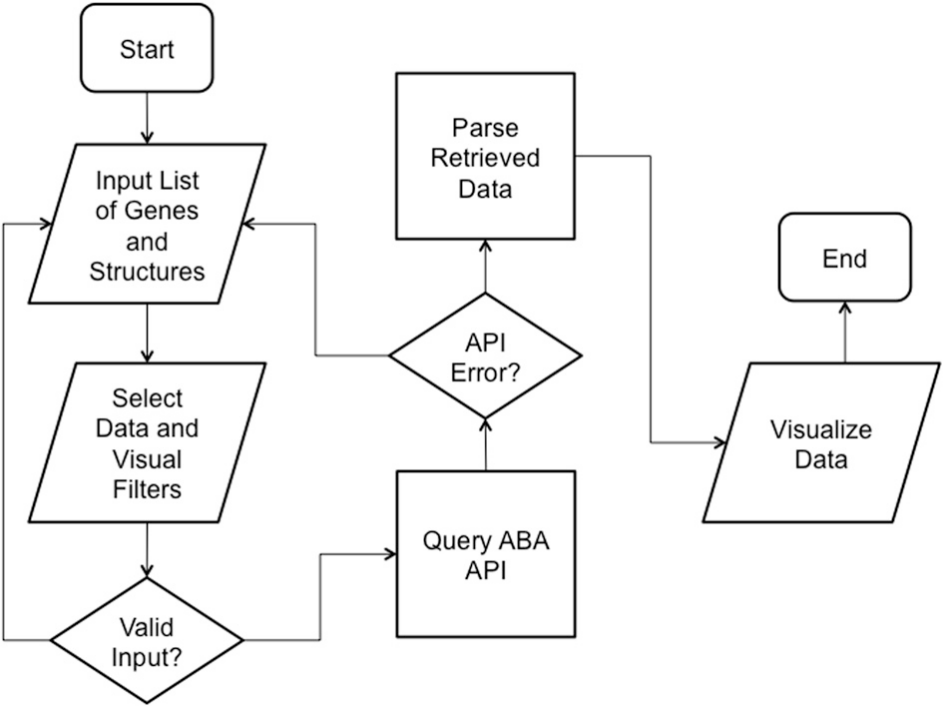
**ABADV Flowchart**. Depicts how each process in ABADV works, as well as the flow of these processes. The user sets filters (probe and section), visualization type (pie, bar, heatmap, or all three) and inserts a list of genes and brain structures of interest. Upon validating the input, ABADV queries the ABA API for expression data. If the query successfully returns data, ABADV will utilize D3 to display the results.

We implemented ABADV to selectively display expression energy across many genes and brain structures at once. The flowchart in Figure 1 depicts the program flow from user input to visualization.

**Figure 2:**
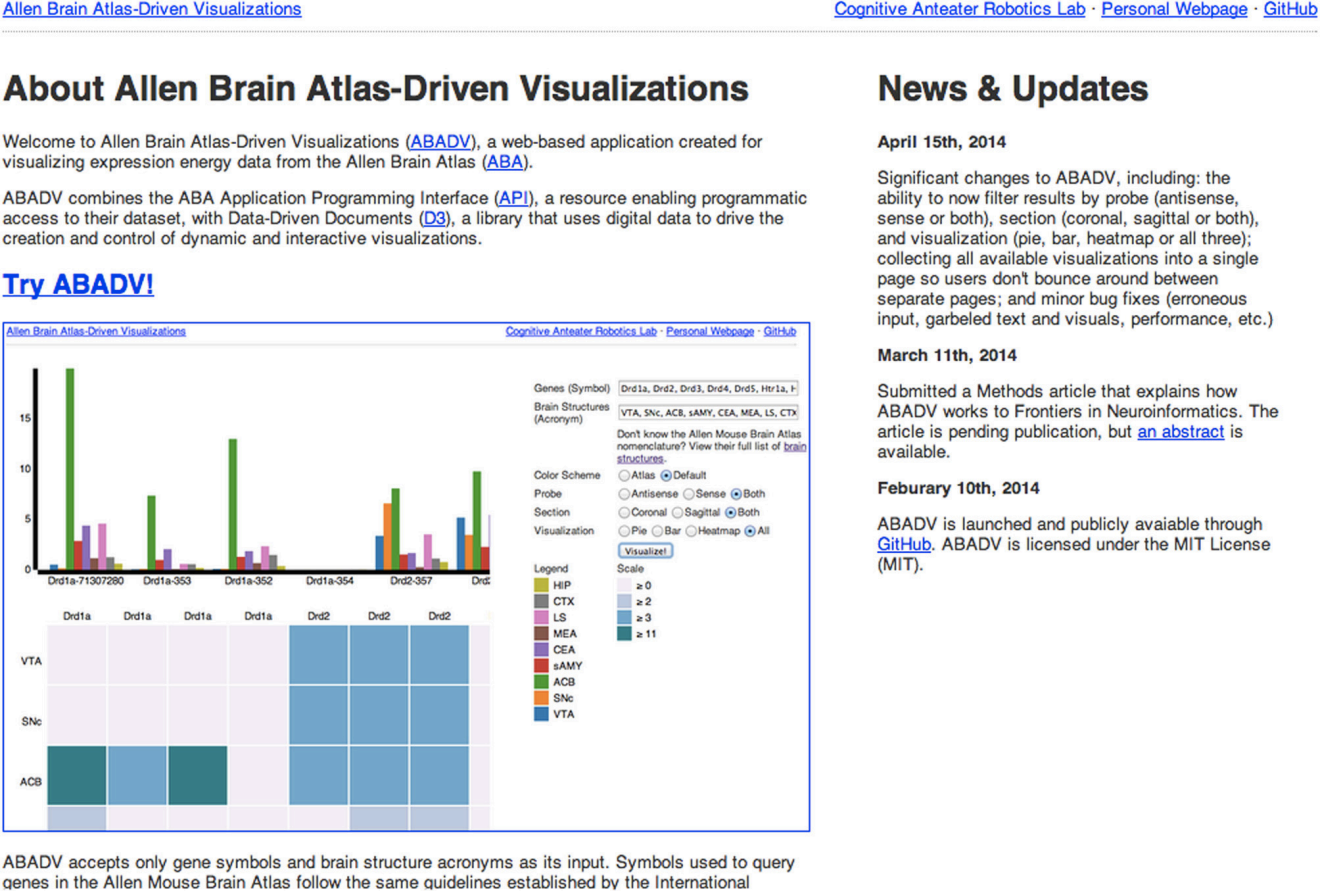
**Screenshot of ABADV Main Page**. A screen capture of the main page for ABADV.

**Figure 3:**
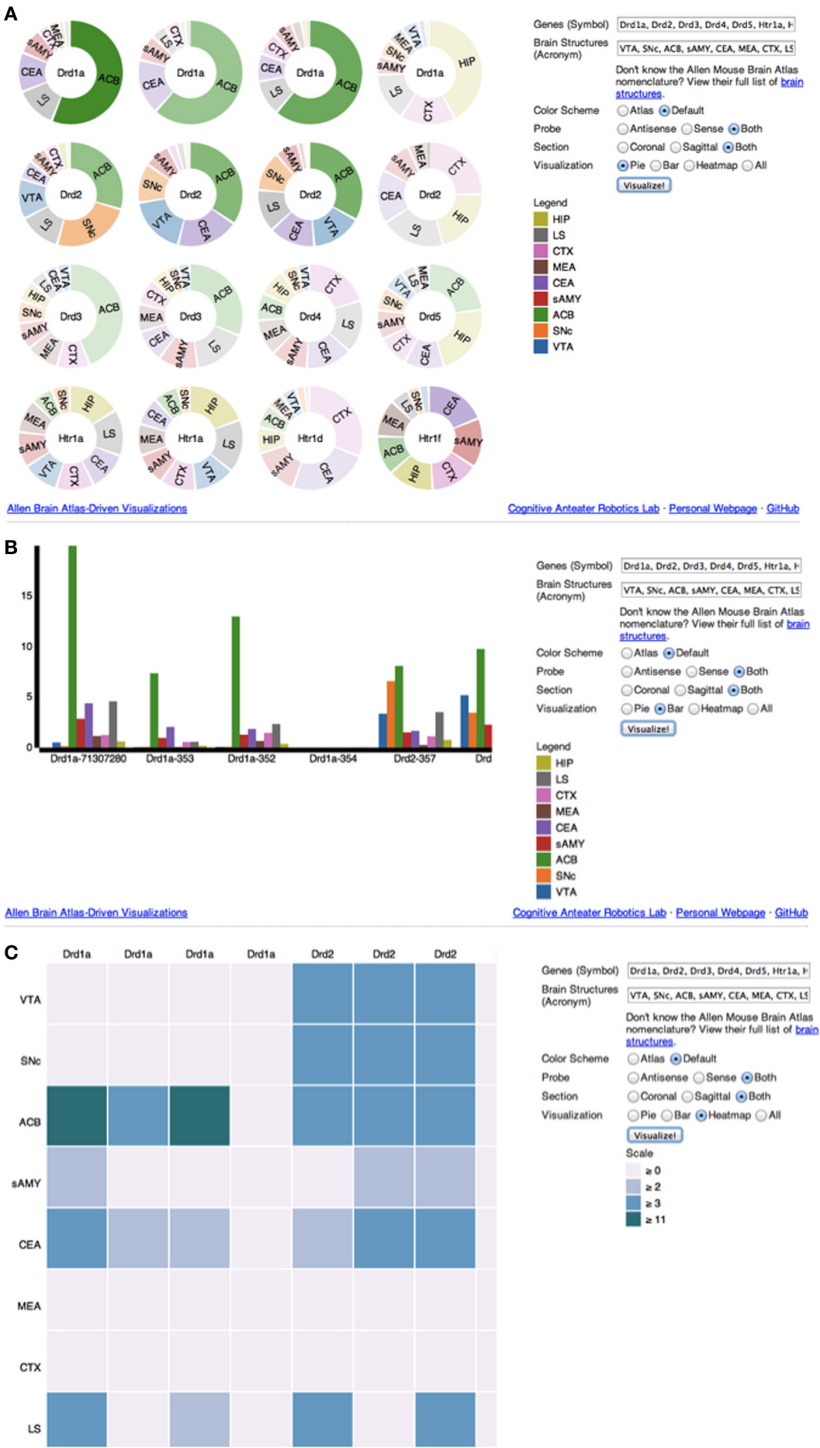
**(A–C) Screenshot of ABADV Visualizations**. A screen capture of each visualization page for ABADV. Users can select different filters and type in a list of genes and brain structures of interest. Depending on the visualization, the option to choose a color scheme is provided. For each plot, users can hover their mouse pointer over a particular portion of a graph to pull up its underlying expression energy value. **(A)** Pie Chart. **(B)** Bar chart. **(C)** Heatmap.

Upon accessing the web application (Figure 2), the user is provided with several options to filter and visualize results (Figures 3A–C). For filter options, the user can set which type of probe (antisense, sense or both) and section (coronal, sagittal or both) to retrieve. These choices are provided to the user because different probe features and brain sections may influence a gene's expression profile. For visualization options, the user can either select multiple pie charts, grouped bar charts, a heatmap or all three. By default, ABADV is set to visualize all three options. After selecting these filters and visualizations, the user can type a list of genes and brain structures. The web application accepts gene symbols and brain structure acronyms. Symbols used to query genes in the ABA follow the same guidelines established by the International Committee on Standardized Genetic Nomenclature for Mice (Eppig et al., 2012). A list of gene symbols can be downloaded at http://www.informatics.jax.org/genes.shtml (Eppig et al., 2012). Acronyms used to query brain structures in the ABA are based on the Allen Reference Atlas. These brain structure acronyms can be found at http://atlas.brain-map.org. ABADV also provides a full list of these structures, located in a hyperlink below the text field where users insert brain structures (Figures 3A–C).

After the user has entered their genes and brain structures of interest, they are ready to click the “Visualize!” button, which triggers the application to query the ABA API. Before accessing the API, ABADV ensures that the user's inputs are valid, prompting the user to start over should they not type appropriate search terms. If inputs are valid, ABADV will query the API. During this process, ABADV begins by querying the API with every gene listed to obtain a set of SectionDataSetId values. A SectionDataSetId is a value associated with the collection of images and metadata for a gene expression experiment. Some genes may return multiple SectionDataSetId values, while other genes may return null. These SectionDataSetId values are necessary to access expression energy values. Once a list of SectionDataSetId values has been obtained, the application completes its query, using each SectionDataSetId and brain structure to acquire expression energy values. Expression energy values are retrieved in JSON, which is compatible with D3, making JSON an ideal format for data interchange. Should any error arise from attempting to access the API in this process, ABADV will prompt the user to try again.

With data retrieved from the API and stored in the user's web browser, ABADV can now visualize this information to the user. Depending on the type of visualization the user selected (multiple pie charts, grouped bar charts, heatmap, or all three), the data must be further parsed and formatted so that it is compatible with the user's visualization choices.

If multiple pie charts were selected, ABADV will use the appropriate D3 features to generate pie charts, one pie chart for each gene of interest with a pie slice within a chart representing a brain structure of interest (Figure 3A). Multiple pie charts are useful for demonstrating relative proportions. To quickly identify prevalent genes from their query, ABADV augments the color intensity of the pie charts such that genes with lower expression energy relative to other genes from the query will appear more transparent, while genes with higher expression energy will appear more opaque. Furthermore, the width of a pie slice represents the total amount of expression energy of a given gene in a brain structure relative to other brain structures within the same gene.

If grouped bar charts were selected, ABADV will appropriately create various bar charts of expression energy (Figure 3B). Grouped bar charts provide a way to show information about different sub-groups (brain structures) of the main categories (genes). Each bar within a group, categorized by gene of interest, represents the amount of expression energy found in a brain structure of interest and are colored differently to distinguish between them. The height of the bar is proportional to the amount of expression energy. As such, there is no need to augment the color intensity of each bar, as its height identifies predominant genes.

Lastly, if heatmap was selected, ABADV will substitute each individual gene expression value into a 2D data matrix of colors (Figure 3C). Heatmaps are useful for finding high and low values, as well as patterns. Each row in this data matrix represents a brain structure and each column represents a gene of interest. Different shades of colors per cell represent the actual expression energy value of a gene per structure, with a darker shade denoting high expression energy and a lighter shade denoting low expression energy.

For all three visualizations, ABADV can link back to the ABA experiment page by clicking on any pie chart, bar chart, or cell in heatmap so users may view a summary of its experimental detail, which includes an interactive 3-D representation of gene expression, a histogram of expression energy across major brain structures, probe and gene metadata, and an interactive image viewer that displays ISH images from the experiment. In addition, different color schemes are provided for pie and bar charts. Users can either select a color scheme based on the RGB values assigned to each brain structure according to the anatomic ontology derived from the Allen Reference Atlas (Dong, 2008), or a default categorical color set generated by D3.

### Demonstrations and comparisons

Using the ABA API with D3, we designed ABADV to aid users in visualizing expression energy data across many genes and brain structures. This section presents an example of the type of output ABADV is capable of generating in comparison to the Brain Explorer, an application developed by the Allen Institute for visualizing expression data. These examples were performed using the ABADV web application available at: http://www.socsci.uci.edu/~jkrichma/ABADV/

### Web-application demonstration

For this example, we performed a query comprised of dopamine receptors (*Drd1a*, *Drd2*, *Drd3*, *Drd4*, *Drd5*), serotonin receptors (*Htr1a, Htr1b, Htr2a, Htr2b, Htr2c*), and various brain structures involved in reward: prelimbic area (PL), infralimbic area (ILA), hypothalamus (HY), hippocampal formation (HPF), striatum-like amygdalar nuclei (sAMY), nucleus accumbens (ACB), ventral tegmental area (VTA), and dorsal raphe nucleus (DR). Together, they form a significant part of the reward circuit, a network responsible for processing various aspects of positive emotional stimulus (Nakamura, 2013; Russo and Nestler, 2013). This reward circuit is key for incentive-based drives and goal-directed behaviors (Berridge and Robinson, 1998). Recent findings suggest that, in particular, the dopaminergic neurons in the VTA projecting to the ACB are principally involved in guiding attention toward rewards and consuming rewards (Koob and Le Moal, 2008). It has also been argued that serotonin neurons in the DR have possible functions in reward processing as it interacts with dopamine (Nakamura, 2013). Providing a full overview of reward circuits in the brain is beyond the scope of this manuscript. However, these genes and brain structures associated with the reward circuit help demonstrate the capabilities of ABADV.

Our query returned 22 unique experiments, 12 from dopamine receptor genes and 10 from serotonin receptor genes. Their SectionDataSetId number denotes each experiment, which was truncated to their first three digits for visualization purposes.

**Figure 4:**
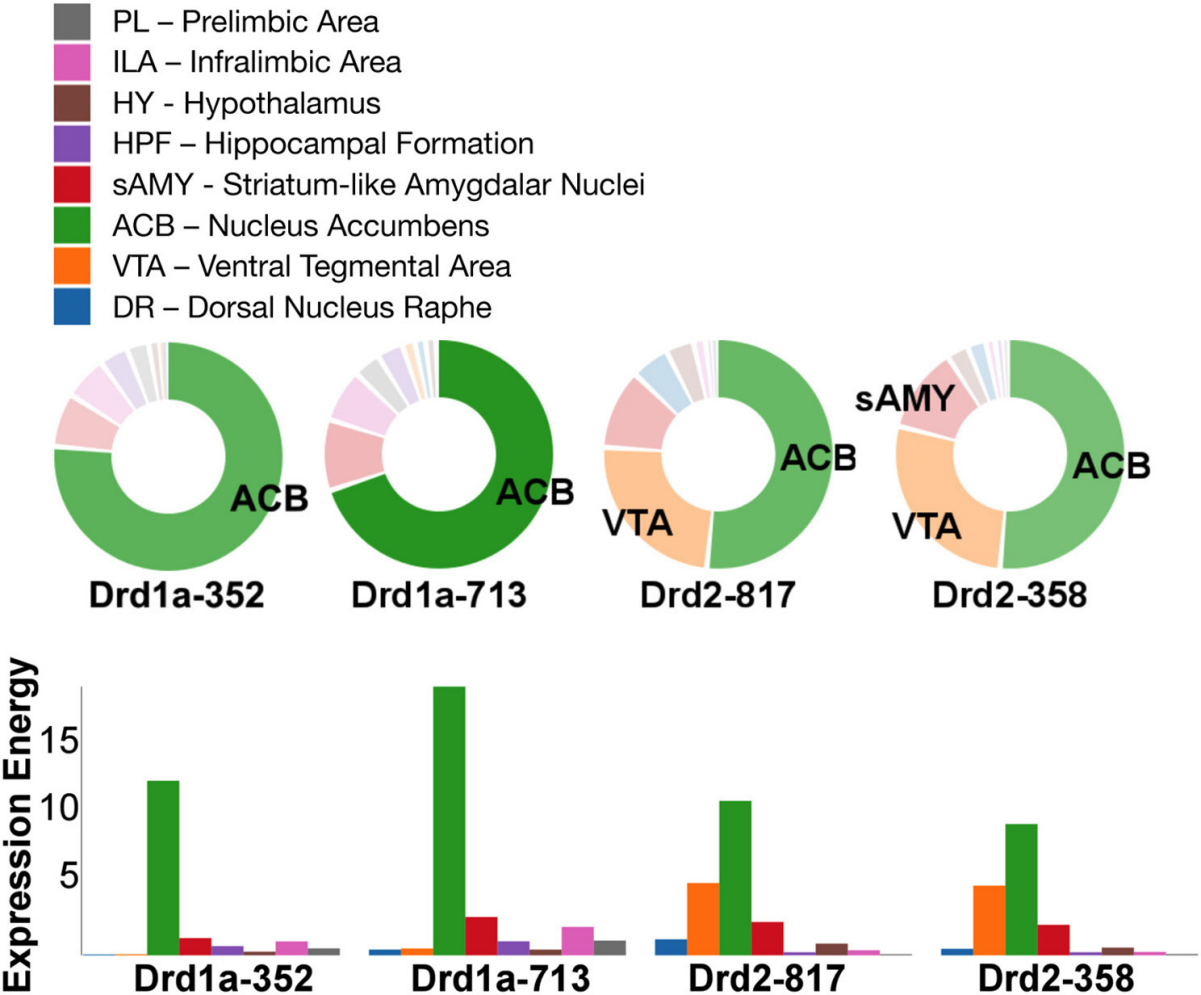
**ABADV Pie and Bar Chart of Dopamine Receptor Genes in Brain Structures Associated with Reward Processing. (Top)** Each pie represents a different gene, while the slices within a pie represents a different brain structure. Opacity of each pie slice denotes the amount of expression energy relative to other gene expression experiments. Size of pie slice denotes the amount of expression energy relative to other brain structure within the same gene. Color scheme derived from both the Allen Reference Atlas or generated by default in D3. **(Bottom)** Each group of bars represents a different gene expression experiment, while each bar within a group represents a different brain structure. Height of bar denotes the amount of expression energy. Default color generated D3 was used in this plot.

**Figure 5:**
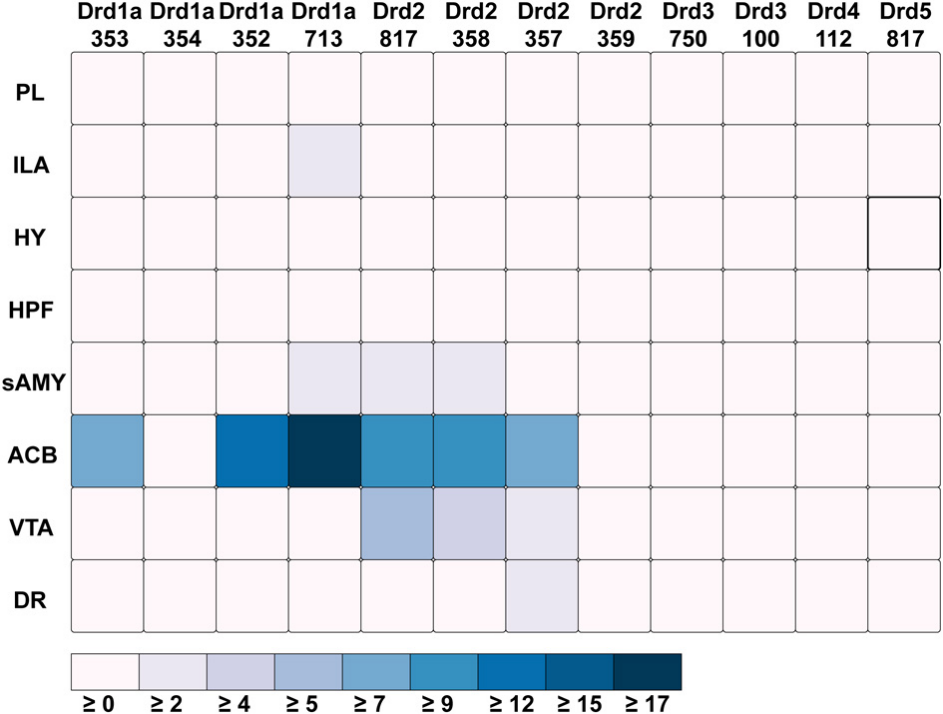
**ABADV Heatmap of Dopamine Receptor Genes in Brain Structures Associated with Reward Processing**. Each cell in the matrix represents an expression energy value for a particular experiment (column) and brain structure (row). The shade of the color denotes how much expression was found in that cell, where the darker the color, the higher the expression, and the lighter the color, the lower the expression.

Out of all the dopamine receptor genes queried, *Drd1a* SectionDataSetId #713 contained the highest amount expression energy, denoted by both the opacity of its slices and height of its bars (Figure 4). Within *Drd1a* SectionDataSetId #713, ACB was highest in expression energy at approximately 20, while expression energy in other brain structures ranged from moderate to nearly quiescent. The heatmap shown in Figure 5 further illustrates this difference in expression, both across brain areas and gene expression experiments. Across other dopamine receptor genes, expression energy in ACB remained dominant, again as denoted by the dark blue shaded cells in Figure 5. However, the total amount of expression found within each gene compared to *Drd1a* SectionDataSetId #713 was lower, as was also shown by its low bar height in Figure 4.

**Figure 6:**
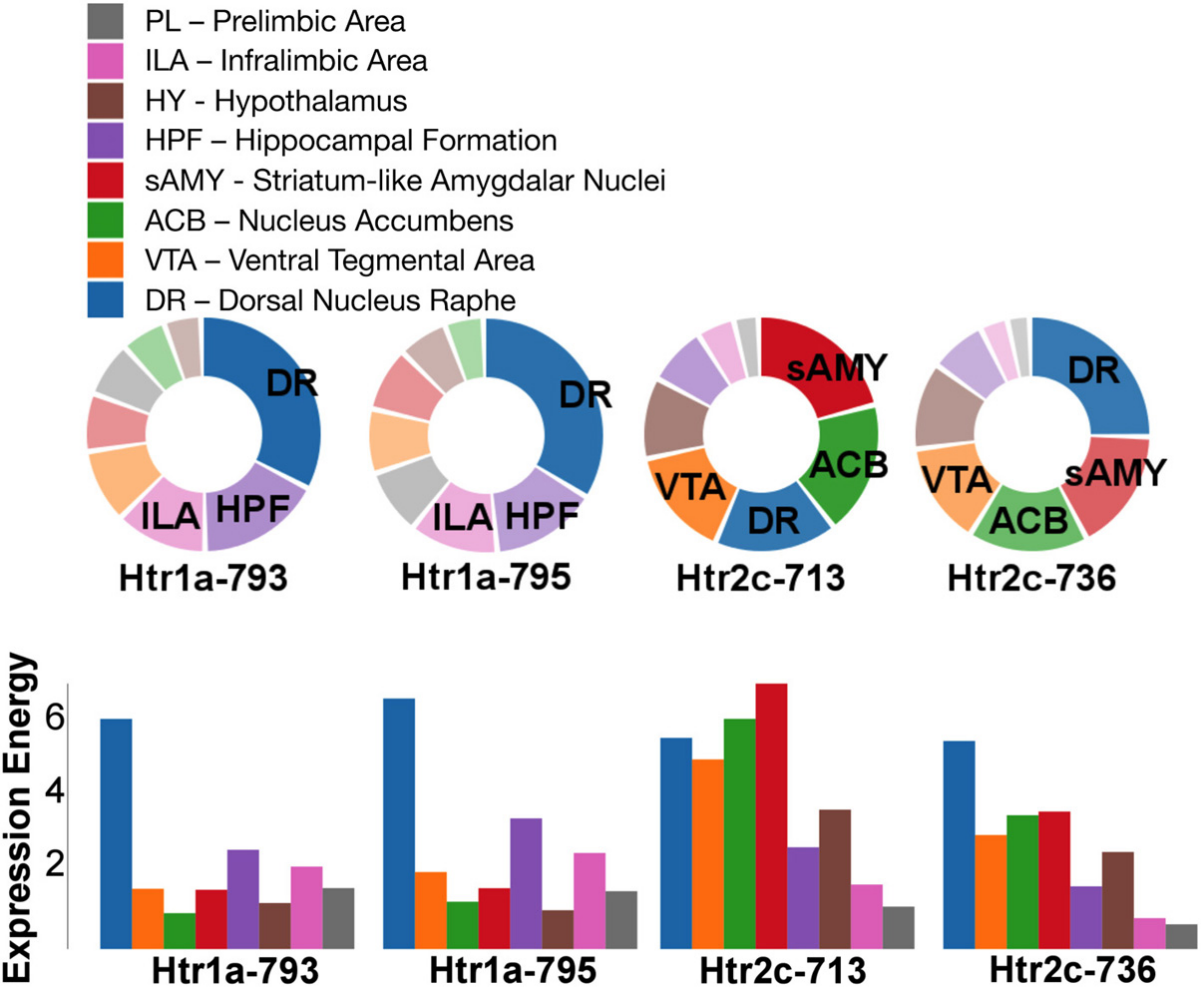
**ABADV Pie and Bar Chart of Serotonin Receptor Genes in Brain Structures Associated with Reward Processing**. **(Top)** Each pie represents a different gene expression experiment, while the slices within a pie represents a different brain structure. Opacity of each pie slice denotes the amount of expression energy relative to other genes. Size of pie slice denotes the amount of expression energy relative to other brain structure within the same gene. **(Bottom)** Each group of bars represents a different gene expression experiment, while each bar within a group represents a different brain structure. Height of bar denotes the amount of expression energy. Default color generated D3 was used in this plot.

**Figure 7:**
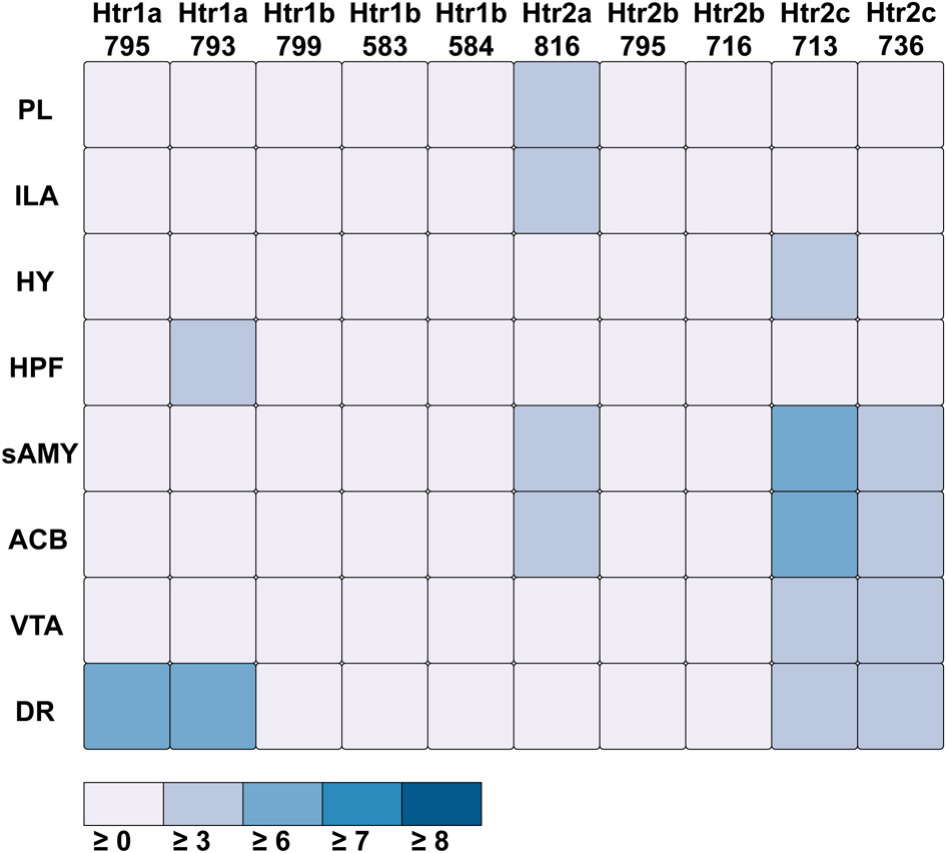
**ABADV Heatmap of Serotonin Receptor Genes in Brain Structures Associated with Reward Processing**. Each cell in the matrix represents an expression energy value for a particular experiment (column) and brain structure (row). The shade of the color denotes how much expression was found in that cell, where the darker the color, the higher the expression, and the lighter the color, the lower the expression.

For serotonin receptors, two particular experiments stood out the most: *Htr1a* SectionDataSetId #793 and *Htr2c* SectionDataSetId #713. These two genes were highest in expression energy values compared to the rest, though where that expression was located at differed between the two (Figures 6, 7). Within *Htr1a* SectionDataSetId #793, expression energy was found highest in DR, denoted by its tall blue bar and large blue pie slice in Figure 6. However, within *Htr2c* SectionDataSetId #713, expression energy was found highest in sAMY, shown in Figure 6 by having the tallest red bar and largest red slice, followed by ACB and then DR. The remaining serotonin receptor expressions were lower, which is depicted by the amount of light shades cells vs. dark shaded cells in Figure 7.

*Drd1a* SectionDataSetId #354 and *Drd2* SectionDataSetId #359 displayed peculiarly low amounts of expression energy across all brain structures (Figures 4, 5). Upon inspection of their respective experimental page in the ABA (via clicking on the pie or chart of these SectionDataSetIds), we realized those two experiments utilized labeled sense RNA probes, while other genes in our query used labeled antisense RNA probes. A sense probe is a strand of RNA that has the same sequence as its target mRNA, while an antisense probe is an RNA strand that is complementary to the sequence of its target mRNA. As such, a sense probe gives a measure of non-specific probe binding due to the chemical properties of the probe, as opposed to an antisense probe which measures its target mRNA. Given that, these sense probes are used to gauge protocol efficacy. A low-to-no expression energy found in sense probes, as we witnessed in our results from *Drd1a* SectionDataSetId #354 and *Drd2* SectionDataSetId #359, assures that any signal detected by its antisense probe (the remaining experiments from our query) is due to sequence-specific binding to mRNA and not with other targets within the cell. The ability to retrieve experimental details directly from the ABA allowed us to unveil this information about our results that would otherwise cause confusion in data interpretation.

### Brain explorer comparison

ABADV complements the Brain Explorer, which is provided by the Allen Institute. Brain Explorer is a free-to-download desktop application for viewing brain anatomy and gene expression data in 3D (Lau et al., 2008). It is integrated with the ABA, enabling users to view spatially registered gene expression data in 3D at a 200-μ m^3^ resolution. With Brain Explorer, users have the ability to display ISH expression data from multiple genes superimposed on each other in 3D, as well as fully interact with the Allen Reference Atlas. To obtain gene expression data for display in Brain Explorer, users can either perform a search on their website at http://mouse.brain-map.org/, select gene(s) of interest, then click on the “View in 3D” link in the search results list or search within Brain Explorer itself.

**Figure 8:**
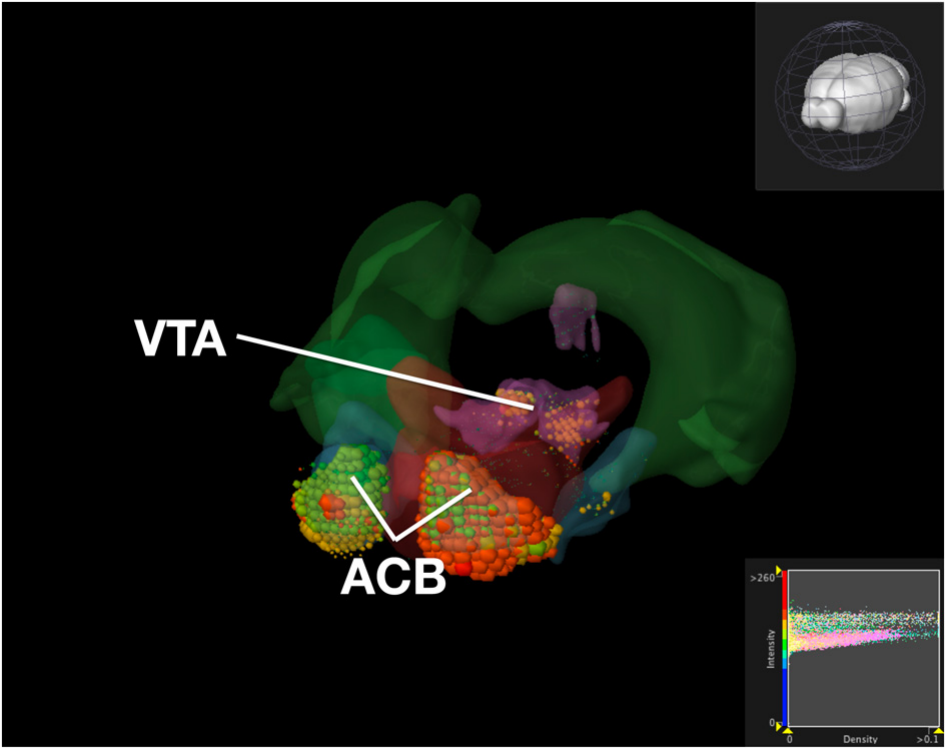
**Expression of Dopamine Receptor Genes in Brain Structures Associated with Reward Processing Using Brain Explorer 2**. Colored spheres represent the amount of expression in a given brain structure, where blue–green is low, yellow is medium, and red is high. Spheres also represent each 200-μ m^3^ voxel, with the size of the spheres directly proportional to the number of objects detected in each voxel.

**Figure 9:**
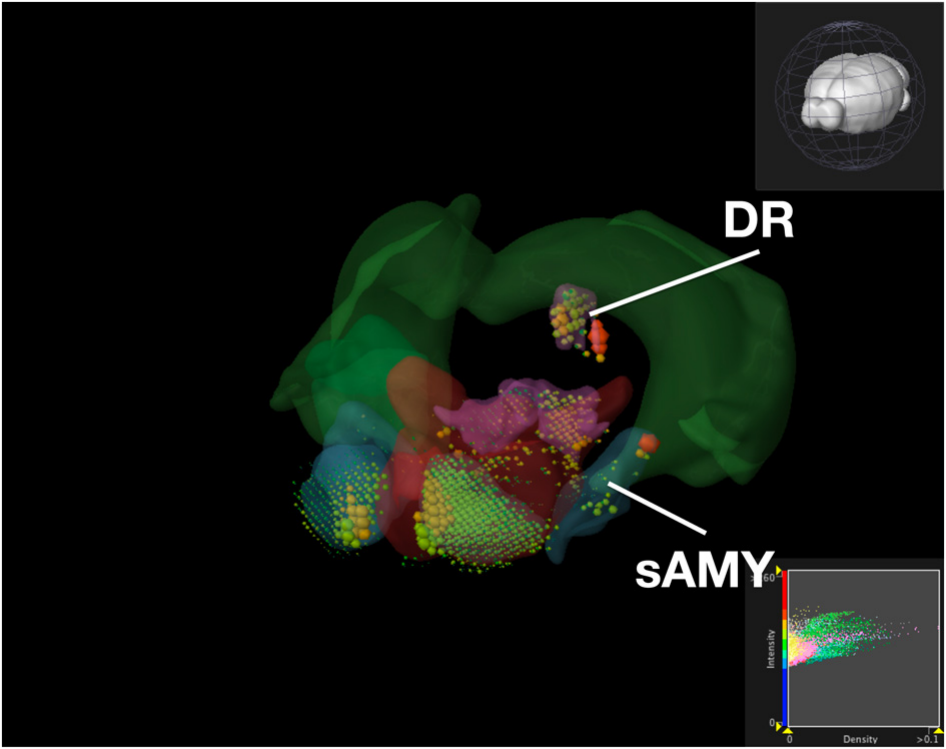
**Expression of Serotonin Receptor Genes in Brain Structures Associated with Reward Processing Using Brain Explorer 2**. Colored spheres represent the amount of expression in a given brain structure, where blue–green is low, yellow is medium, and red is high. Spheres also represent each 200-μ m^3^ voxel, with the size of the spheres directly proportional to the number of objects detected in each voxel.

To compare our visualization with Brain Explorer, we performed the same query as we did with ABADV using genes that encode dopamine receptors and brain structures associated with the reward circuit (see Web-Application Demonstration section). We first searched for our genes of interest through their website. After downloading each gene into Brain Explorer, we navigated through their structural ontology panel within the application, turning on all 3D polygonal brain structures associated with the reward circuit and turning off all other brain structures. Figure 8 is a screenshot of Brain Explorer's main window application displaying all 12 dopamine receptor genes and 8 brain structures from our query. Figure 9 is a screenshot of Brain Explorer's main window application displaying all 10 serotonin receptor genes and 8 brain structures from our query. Colored spheres indicate the amount of expression energy in a given brain structure, where blue–green is low, yellow is medium, and red is high. Spheres are used to represent each 200-μ m^3^ voxel, with the size of the spheres directly proportional to the number of objects detected in each voxel.

While the results from Brain Explorer are identical to ABADV because the data is the same, there are complementary differences. As with our dopamine receptor gene query using ABADV (Figures 4, 5), the results from Brain Explorer revealed ACB containing the highest amount of expression energy compared to other brain structures, as denoted by the dense, bright-colored voxels located on the rostral end of the visualized mouse brain in Figure 8. Likewise, compared to our results using ABADV to retrieve serotonin receptor genes expression (Figures 6, 7), the results from Brain Explorer also revealed both DR and sAMY were highest amongst all other brain structures in expression energy values, which is demonstrated by the large, localized voxels in the posterior and lateral end of the visualized mouse brain in Figure 9.

Superimposing multiple genes using Brain Explorer made it difficult to identify which dopamine or serotonin SectionDataSetId were expressing the most. In order to know which SectionDataSetId expressed the most in Figures 8, 9, in Brain Explorer you can either click on the individual voxel to reveal its metadata, or selectively turn off the display of other SectionDataSetIds and view each experiment separately. You can also control expression visibility by setting thresholds on expression values (Figures 8, 9, bottom right corner). In ABADV, however, comparisons between genes are easier to portray because a pie chart, bar chart, or heatmap cell is generated for each gene, which does not get in the way of one another.

Another key difference between Brain Explorer and ABADV is the type of expression data presented. In Brain Explorer, clicking on colored spheres reveals the expression density and intensity of that voxel, whereas ABADV displays expression energy across the entire brain structure. While the Brain Explorer offers detailed expression data at the voxel level, the ABADV allows quantitative analysis of expression energy across the entire brain structure.

Aside from these key advantages of ABADV, Brain Explorer does offer features not available in ABADV. In particular, Brain Explorer enables 3D representation of gene expression with the original full resolution 2D tissue sections is a rich feature that may make data exploration convenient, easily mapping the localization of expression. By selecting a colored voxel like those in Figures 8, 9, Brain Explorer can display the name of the structure and its location in its atlas ontological hierarchy while simultaneously displaying its original image data that was used to generate these quantitative results. Thus, the ABADV and Brain Explorer are complementary.

## Discussion

ABADV, which is available for use at: http://www.socsci.uci.edu/~jkrichma/ABADV/, has been developed to extend the ABA by providing users with a quick and intuitive way to survey large amounts of expression energy data across multiple brain regions of interest. ABADV combines the ABA API with D3 to obtain and visualize expression energy data from various genes and brain structures using pie charts, bar charts, and heatmaps to display these quantified measurements. Our demonstration of querying the ABA for available dopamine and serotonin receptor genes showed that ABADV could help identify prevalent genes and brain structures that may otherwise be obscured in other types of visualizations, as we showed in our Brain Explorer example where their application superimposes data on top of one another. Using ABADV revealed heavily expressed brain regions such as the nucleus accumbens and dorsal raphe across dopamine and serotonin receptor genes, respectively, (Figures 4–7). This quantified, exploratory analysis makes it easier for users to obtain such results without having to delve deep into the intricacies of the ABA. ABADV serves as a complement to the resources provided by the ABA and should be used in conjunction with other data sets and techniques for complete analysis.

Future work may involve incorporating other forms of visualizations and different data sets onto ABADV. ABADV was designed in such a way that someone with some web technology experience could modify the existing code to extend its capabilities. The source code can be obtained at: http://github.com/UCI-CARL/ABADV. Thus, ABADV is not limited to these summary-level visualization options we have provided. Presently, ABADV does not offer the same richness the ABA provides nor can it presently correspond with other resources available through the ABA. In the future, ABADV will be extended to include other neuroinformatic resource, such as the Allen Human Brain Atlas and the Allen Developing Mouse Brain Atlas, and also resources outside of the ABA such as Gene Expression Omnibus (Edgar et al., 2002) and ArrayExpress (Parkinson et al., 2011). With big data science emerging, it is paramount that we build tools like ABADV alongside in order to effectively mine these growing resources and make great leaps in neuroscience discovery.

### Conflict of interest statement

The authors declare that the research was conducted in the absence of any commercial or financial relationships that could be construed as a potential conflict of interest.
